# Test-reduced teaching for stimulation of intrinsic motivation (TRUST): a randomized controlled intervention study

**DOI:** 10.1186/s12909-024-05640-7

**Published:** 2024-07-03

**Authors:** Theresa Faure, Imke Weyers, Jan-Bennet Voltmer, Jürgen Westermann, Edgar Voltmer

**Affiliations:** 1 Institute for Social Medicine and Epidemiology, University of Lübeck, Ratzeburger Allee 160, 23562 Lübeck, Germany; 2 Institute for Anatomy, University of Lübeck, Ratzeburger Allee 160, 23562 Lübeck, Germany; 3 Department of Psychology/Social Psychology, Distant-Learning University (FernUniversität in Hagen), Universitätsstraße 47, 58097 Hagen, Germany

**Keywords:** Medical students, Randomized controlled study, Stress, Affect, Self-efficacy, Motivation, Academic performance, Friendly feedback

## Abstract

**Background:**

The anatomy dissection course is a major part of the first two years of the traditional medical curriculum in Germany. The vast amount of content to be learned and the repeated examination is unanimously perceived by students and teachers as a major stress factor that contributes to the increase of psychosocial stress during the first two years of the course of study. Published interventions for specific stress reduction are scarce.

**Methods:**

In a randomized, controlled design two intervention groups were compared with a control group (CG) over the whole dissection course (nine measuring points before, during and after first and second semester). The ‘Stress Management intervention (IVSM)’ targeted at the setting of personal standards, the ‘Friendly Feedback intervention (IVFF)’ at the context of frequent testing. Quantitative surveys were distributed at nine measuring points. The questionnaire comprised validated instruments and self-developed items regarding stress, positive and negative affect, anxiety, intrinsic and extrinsic motivation, self-efficacy, and perceived performance.

**Results:**

Out of 195 students inscribed in the dissection course, 166 (85%) agreed to participate in the study. The experience of stress during the dissection course was significantly higher in the CG than in the IVFF. Anxiety and negative affect were lower in students of the IVFF while positive affect, intrinsic motivation, and self-efficacy were higher than in the CG. For anxiety and negative affect in the IVSM this was especially seen at the end of the second semester. The self-perceived increase in both knowledge and preparedness for the first big oral and written examination did not differ between the study groups. About three quarters of the participants would choose the intervention ‘Friendly Feedback’ if given the choice.

**Conclusions:**

Replacing formal tests with friendly feedback has proven to be an effective measure to reduce stress and negative affect and foster positive affect, self-efficacy, and intrinsic motivation, while it did not impair self-perceived academic performance.

**Supplementary Information:**

The online version contains supplementary material available at 10.1186/s12909-024-05640-7.

## Background

Medical education has a worldwide reputation of being stressful [1–3]. In Germany, decreasing proportions of students with a healthy behavior and experience pattern and increasing proportions with risk patterns for overexertion and burnout as well as symptoms of anxiety and depression have been reported especially for the first two years before the first major exam [1, 4, 5]. Performance pressure in the face of frequent testing, a fear of bad grades, and the vast amount of content to be learned are reported as some of the main reasons for stress [6, 7].

In Germany, medical studies courses last for six years. In regular programs, after two years a first major exam has to be passed (M1/formerly Physikum, a centrally organized state exam, identical for every (participating) university). Major topics of the first two years with a vast amount of content to be learned include anatomy, biochemistry, and physiology, which are often presented together in the third and fourth semester. At the university of Lübeck, in a first attempt to reduce students’ stress, anatomy has been rescheduled to the first and second semester. In the dissection course, students have to pass an oral examination every week in both semesters. If they fail, they have to retry the examination. The content taught and the mode of examination are practiced in a comparable way in most standard medicine courses in Germany. In some faculties an exam is written at the end of the semester instead of, or in addition to, the tests during the semester. Students are informed that 95% pass the examination. However, the vast amount of content to be learned and the repeated examination is unanimously perceived by students and teachers as a major stress factor that contributes to the increase of psychosocial stress during the first two years, as seen in the results of the ongoing longitudinal study at Lübeck university (Luebeck University Students Trial, LUST) [4, 5]. We chose the dissection course in anatomy for the study because at the university of Lübeck it is the first of the three major subjects that students find particularly stressful in the first two years (preclinical phase). The aforementioned transfer of the course to the first year of study also demonstrates the interest of the Institute of Anatomy (whose director is also the Dean of Medical Studies) in health promotion and stress reduction. We also intended to use the results in anatomy for a comparable approach in the other two stressful topics (biochemistry, physiology) of the preclinical phase.

According to self-determination theory (SDT), frequent testing is a characteristic of a controlling teaching style that may foster extrinsic motivation and impair intrinsic motivation among students [8, 9]. According to this model, a teaching style that fosters autonomy and intrinsic motivation in students is characterized by considering and taking interest in the students’ perspective, positive feedback and appreciation, as well as explaining fully the demands and employing a blame-free communication [10, 11]. Comparative studies showed that an autonomy-promoting teaching style yielded better results in conceptual learning, and long-term memory, along with reducing stress, than a controlling style [12, 13]. It was also positively correlated with vitality and well-being and negatively with feelings of depression or burnout. In contrast, a controlling teaching style was correlated with negative affect and burnout [14]. Integrated model analyses in students also found a strong relationship between intrinsic motivation and fulfilled basic needs and positive affect and self-efficacy [15, 16]. Positive affect and self-efficacy were in turn correlated positively with engagement and academic success [16–18]. In contrast, negative affect was correlated with increased stress and lower academic performance [19]. While in a meta-analysis stress in general or related to academia obtained the largest but still small correlations with academic success, students who felt that stress affected their performance had lower academic success and reported more stress [20].

While tests and exams belong to the external or structural context of a course of study, it should be noted that often high-achieving students tend to cause stress for themselves by setting their personal standards very high [21]. In particular, maladaptive forms of perfectionism with constant self-criticism and a fear of being judged by others were correlated with stress, anxiety, and depressive symptoms as well as test anxiety [22]. In a study of medical students, maladaptive perfectionism was consistently associated with distress and symptoms of depression as well as hopelessness regarding academic performance [21]. Increasing perfectionism was also correlated with declining resilience in medical students [23]. A high performance-based self-esteem was correlated with emotional exhaustion in a student group with this characteristic [24]. This may be especially harmful under conditions of a controlling teaching style [25].

Most of the published interventions addressing stress in the anatomy dissection course focus on the issue of dissecting human bodies or stress in general [26–29]. In this study, we compared two interventions in the dissection course aimed at reducing self- or context-generated stress and fostering intrinsic motivation to a control condition with regular teaching and testing. The ‘Stress Management intervention (IVSM)’ is a mindset-oriented program (addisca training) that was targeted at the setting of personal standards. In a pilot study, effects were particularly evident during periods of testing [30]. The ‘Friendly Feedback intervention (IVFF)’ was based on SDT and comprised principles of autonomy-supportive teaching [8, 31]. It was targeted especially at the context of frequent testing (see method section). The research question was: How do the interventions influence the outcome parameters (mental health, self-efficacy, intrinsic/extrinsic motivation, and perceived status of preparation for the first major exam) compared to the control group with the standard program? Our hypotheses, based on reported effects for both interventions, were that in both intervention groups (a) the perception of stress would be lower (in the IVSM particularly at the end of a semester), (b) self-efficacy and intrinsic motivation would be higher than in the control group, while (c) there would be at least no difference in the perceived status of preparation between all study groups.

## Methods

### Interventions and control

Using a randomized, controlled design, two intervention groups were compared with a control group over the whole dissection course (first and second semester).

Stress Management intervention (IVSM): The participants of this group underwent a one-day workshop on stress management before the beginning of the dissection course [(addisca training), 32]. The addisca training was initially developed in a clinical context at the Center for Integrative Psychiatry at the University of Lübeck [32] and is based on a metacognitive approach [33]. It has also been successfully applied in non-clinical settings (schools, universities, companies), including students [30]. In this study, students were trained accordingly to deal with unproductive patterns of thinking and in the use of effective learning strategies. As the control group, they were then tested weekly with the option to either pass or fail.

Friendly Feedback intervention (IVFF): The preparation and knowledge of students were checked in a friendly discussion. Feedback was given on whether the preparation seemed satisfactory or further preparation would be recommended. Students were then responsible for how they dealt with the feedback. Based on SDT, the IVFF targeted in particular the attitude of the teachers, focusing on knowledge and not on knowledge gaps, considering students’ perspective, giving feedback on preparation and not on personality, and giving appreciative feedback for good preparation or growth-oriented feedback in the case of gaps [for more detailed description see 14, 34]. Teachers were given instruction on these principles in a preparative session before the start of the dissection course.

Control group (CG): The participants of the control group were tested weekly with the option to either pass or fail.

The first-semester students were personally informed about the study conditions and the voluntary nature of participation in two events before the start of the course. An information sheet was made available online. Students gave their written consent to participate in the study. There were no general exclusion criteria. For the identification of datasets in the longitudinal analyses participants were asked to generate a personal (pseudonymous) identification code and/or provide the matriculation number. The students were informed that the study team had no access to personal data via their matriculation number and that the Student Service Center had no access to the study results.

The participating students were stratified for gender and randomly distributed to the three study conditions. The dissection course was organized in four shifts on two days. A maximum of 51 places were available for students in each shift. To avoid interference effects between the interventions, one shift was reserved for each intervention group. Because there were more than 153 students willing to participate, a slightly larger number of students were randomized to the control group and distributed over two shifts. The randomization was carried out in Excel, using the RAND formula for male and female participants separately. After sorting the participants by random numbers, the same number of participants were assigned to each intervention group up to a maximum of 51, thereby taking the total gender ratio into account. The remaining participants were assigned to the control group.

The evaluation followed a mixed-method approach, with questionnaires being distributed for the quantitative analysis while interviews were used in four focus groups for the qualitative analysis. We report here the results of the main instruments of the quantitative analysis. The detailed report of the qualitative results is beyond the scope of this article and will therefore be provided in a separate article.

### Measures

Questionnaires were presented to the participating students before starting the course (t0 in paper form), on four occasions during the course in the winter semester (t1 – t4), at a follow-up after the winter semester (t5), and at three measurement points during the summer semester (t6 – t8; except for t0, all via QR Code for Lime Survey). During the semesters, the questionnaires were presented just before the start of the dissection course to capture the experiences and feelings connected to the course. The questionnaire comprised the following, validated instruments and self-developed items:

#### Experienced stress

A single-item measure probing the degree of subjective feeling of stress in the previous week has been used in several previous studies and was highly correlated with different measures of emotional distress (‘How would you rate your feeling of stress in the past week?’) [35]. The item was rated on a nine-point Likert scale from 1 - not at all to 9 - very much.

#### Positive and negative affect scales (PANAS)

The Positive and Negative Affect Scales uses 10 adjectives to examine actual mood state with five items for positive affect and five items for negative affect (e.g., ‘active’, ‘anxious’) [36, 37]. Response options are rated on a five-point Likert scale from 1 - not at all, to 5 - extremely. The time frame given was ‘in the last days’.

#### State-trait anxiety inventory (STAI-SKD)

The German version of the STAI-SKD examines the situational feeling of anxiety [38, 39]. The five items (e.g., ‘I am tense’) are scored on a four-point Likert scale from 1 - not at all, to 4 - very much.

#### Self-efficacy (WIRKSTUD)

The study-specific self-efficacy (WIRKSTUD) was examined using seven items (e.g., ‘If I prepare myself sufficiently, I can achieve a good performance in the test/friendly feedback’) [40, 41]. Response scores were rated on a four-point Likert-scale from 1 - not at all to 4 - very much.

#### Learning motivation

As proposed by Brahm et al. [42, 43] intrinsic learning motivation was measured with three items (e.g., ‘I work and learn for my course of study because the learning content interests me’) [44] as well as extrinsic learning motivation (e.g., ‘most important for me is to get good grades in the course of study’) [45]. Items were rated on a six-point Likert scale from 1 – does no apply at all to 6 - does apply exactly.

#### Performance

As surrogate parameters for the final grades in M1, at t5 and t8 we applied two items on the perception of preparation for this first major examination: ‘I feel well prepared for the written/oral part of M1’. Response options were rated on a five-point Likert scale from 1 – do not agree at all to 5 – agree completely. Additionally, the participants were asked to estimate their medical and anatomical knowledge in terms of percentage (0-100%) [46].

#### Personal preference

At the end of each semester (t5 and t8) students were asked, which condition they would have chosen if given the option.

In terms of demographics, age and sex were integrated. A detailed list of which instruments were used at which time-point in the study can be found in the Additional File 1. In the power analysis, we assumed a willingness to participate by at least 150 students, stratified by gender and randomized to about 50 in each of the three study arms. Assuming a mean effect size (*d* = 0.6) in the comparison between the intervention and control group [35] at t5, a power of 80% appeared feasible in the selected study design.

### Ethical approval

The study protocol has been approved by the Ethics Committee of the University of Lübeck (file number: 21–313).

### Statistical analysis

Data analyses were conducted using R and SPSS for Windows, Version 22.0 (IBM Corp., Armonk, NY, USA). In the current study, we collected data across multiple time points on several outcomes of interest. To analyze these data, we used multilevel modeling (MLM) to model the effects of time and other predictors on our outcomes, while accounting for the nonindependence of observations among individuals [47]. Specifically, we included random intercepts for each participant, allowing for individual differences in baseline levels. The intraclass correlation coefficients (ICC) in our study ranged from 0.003 to 0.230, indicating that there was significant within-subject variability that needed to be accounted for in our analyses [48]. By using MLM, we were able to appropriately model this variability and obtain accurate estimates of our effects of interest.

We used linear mixed modeling [49] to investigate the development of the outcome parameters (stress, affect, and anxiety; self-efficacy, motivation; self-perceived performance) over time. Exclusions were limited to participants who were not correctly assigned to one of the three conditions. Using mixed linear modeling, we used all available data points for each participant across different time points, effectively analyzing data from between 132 and 162 participants at each time point. Specifically, we modeled each of the variables described above as an outcome of two predictors, time and study group, as well as the interaction effects between time and study group. This allowed us to test whether the effect of the group on the outcome variables changed over time. If significant interaction effects were detected, we conducted Tukey-adjusted post hoc tests [50] to explore the specific group differences at each time point.

Overall, this analytical strategy allowed us to investigate the development of the outcome variables over time, while accounting for within-subject variability and testing for group differences in the outcome variables. Additionally, the inclusion of interaction effects between time and study group allowed us to examine whether the effect of group on the outcome variables changed over time.

## Results

Out of 195 students inscribed in the dissection course, 166 (85%) agreed to participate in the study. After randomized distribution to the three study conditions, 65 students participated in the control group (CG), 50 in the Stress Management intervention (IVSM), and 51 students in the Friendly Feedback intervention (IVFF).

### Stress and anxiety

**Fig. 1:**
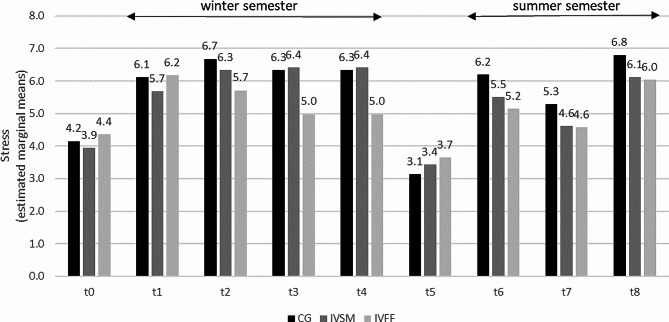
Stress scores of students in three study conditions (single item). CG: control group; IVSM: intervention stress management, IVFF: intervention friendly feedback t0 before, t1 - t4 winter semester, t5 semester break, t6 - t8 summer semester

The experience of stress reported by participants for the previous week was significantly affected by their study group (*F*(2, 163) = 3.09, *p* < 0.05, *ICC*(Model) = 0.409): Specifically, students in the IVFF had an overall lower experience of stress than those in the control group (*t*(167) = 2.45, *p* < 0.05). Moreover, there was a significant interaction effect between time point and study group (*F*(8, 1110) = 3.45, *p* < 0.01). Importantly, however, the difference between the study groups differed between time points as indicated by moderation analysis (*F*(16, 1116) = 3.88, *p* < 0.01): The experience of stress was lower in the IVFF than in the CG at t2, t3, t4, and t6, *ps* < = 0.022, and even lower than in the IVSM at t3 and t4, *ps* < = 0.015 (Fig. 1; Detailed post-hoc comparisons are comprehensively presented in the Additional file 2).

**Fig. 2:**
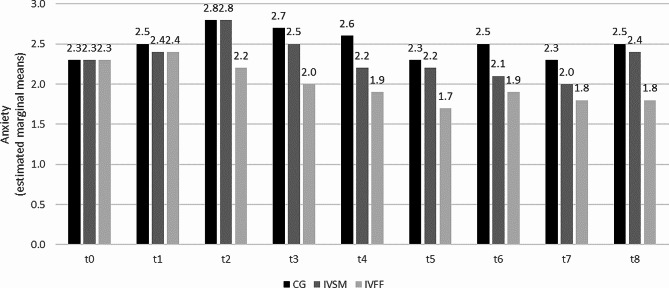
Course-related anxiety (State-trait Anxiety Inventory (STAI-SKD)). CG: control group; IVSM: intervention stress management, IVFF: intervention friendly feedback t0 before, t1 – t4 winter semester, t5 semester break, t6 – t8 summer semester

As with stress, course-related anxiety was significantly affected by the study group (*F*(2, 165.57) = 10.22, *p* < 0.01, *ICC*(Model) = 0.621). Again, students in the IVFF had a lower course-related anxiety than those in the control group (*t*(168) = 4.47, *p* < 0.01). This difference and the significant effect of the measurement timepoint (*F*(8, 1115) = 24.51, *p* < 0.001) were again qualified by an interaction between time and study group: Moderation analysis indicated that study group differed between timepoints (*F*(16, 1115) = 10.22, *p* < 0.01). Anxiety was significantly lower in the IVFF than in the CG at t2 – t8 (*ps* < 0.01). There was also a significant difference between the CG and the IVSM at t4 (*p* < 0.05; Fig. 2; see also Additional file 2).

### Self-efficacy

**Fig. 3:**
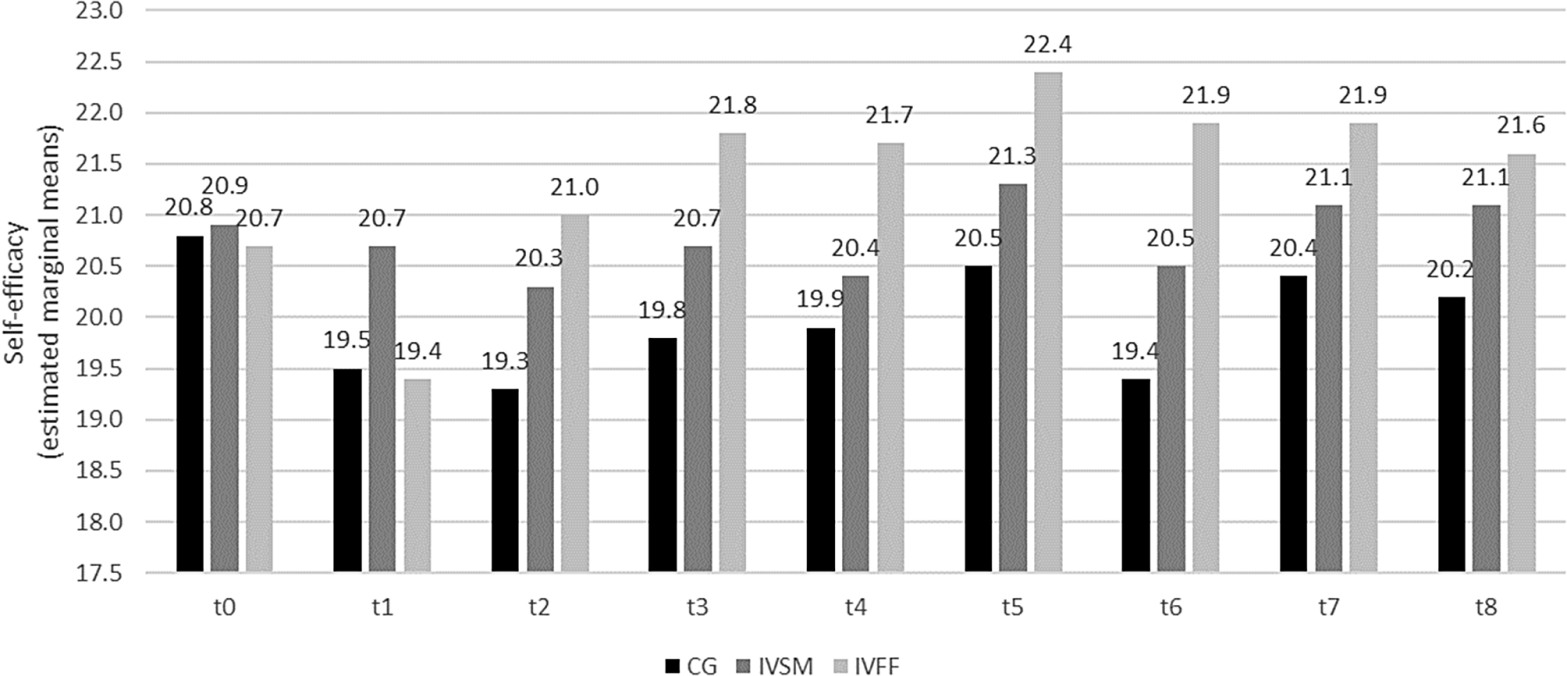
Development of self-efficacy over time (Self-efficacy (WIRKSTUD)). CG: control group; IVSM: intervention stress management, IVFF: intervention friendly feedback t0 before, t1 - t4 winter semester, t5 semester break, t6 - t8 summer semester

Likewise, self-efficacy was also significantly affected by study group (*F*(2, 166.57) = 3.76, *p* < 0.05, *ICC*(Model) = 0.646). Specifically, students in the IVFF had significantly higher scale scores than those in the CG (*t*(168) = -2.69, *p* < 0.05). The effect of study group differed between timepoints (*F*(16, 1111.56) = 3.44, *p* < 0.01): Perceived self-efficacy was significantly higher in the IVFF than in the CG at t2 – t6 (see also Additional file 2). Descriptively all scores of the IVSM were higher than those in the CG but these differences were not significant (Fig. 3).

### Affect and motivation

In contrast to stress, anxiety, and self-efficacy, the differences between study groups regarding positive affect and intrinsic motivation were only qualified by their respective interaction with timepoint: For positive affect, a trend for group differences was visible, with students in IVFF having higher scale scores than those in the CG (*t*(168) = -2.32, *p* = 0.552, *ICC*(Model) = 0.453). More importantly, however, the difference between study groups differed between time points (*F*(16, 1117) = 3.15, *p* < 0.01): Positive affect decreased significantly from t0 to t8 in all study groups. However, the decrease was stronger in the CG than in the intervention groups. Scale scores for positive affect were significantly higher in IVFF than in the CG at t3 and t4 as well as t7 and t8 (*ps* < = 0.02). For negative affect the trend was reciprocal (data not shown; see also Additional file 2).

**Fig. 4:**
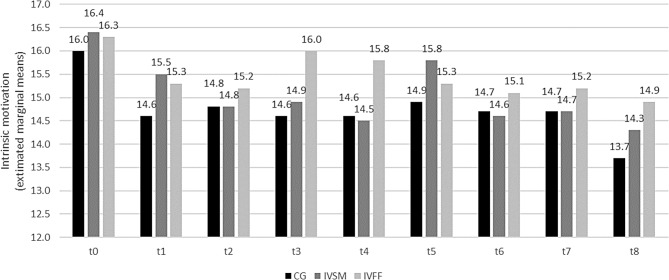
Development of intrinsic motivation (Learning motivation). CG: control group; IVSM: intervention stress management, IVFF: intervention friendly feedback t0 before, t1 - t4 winter semester, t5 semester break, t6 - t8 summer semester

Likewise, the decrease in intrinsic motivation over time (*F*(8, 1105) = 17.10, *p* < 0.001, *ICC*(Model) = 0.554) significantly differed between the study groups (*F*(16, 1105) = 2.32, *p* < 0.05): In post hoc analysis the scores for intrinsic motivation for the IVFF were significantly higher than those for the CG at t3, t4 and t8 (Fig. 4; see also Additional file 2). For extrinsic motivation the differences between study groups were not significant (data not shown).

### Performance

Because there were no gradings in oral examinations and no final written exams at the end of the semesters, we asked the participants at t5 and t8 how they perceived their knowledge in anatomy as well as in all preclinical topics. In addition, students were asked how well prepared they felt for the oral and written part of the M1. From t5 to t8 an increase in perceived knowledge was seen in all study groups (for medical knowledge, *F*(1, 112) = 30.38, *p* < 0.001, for anatomical knowledge, *F*(1, 130) = 39.10, *p* < 0.001). Differences between the intervention groups and the CG were not significant. Likewise, the perceived preparation for the exam increased from t5 to t8 (for the written exam, *F*(1, 120) = 10.96, *p* = 0.001, for the oral exam, *F*(1, 130) = 29.22, *p* < 0.001), but there was no difference in change over time between the study groups (data not shown).

### Preferred condition

At t5 and t8 almost three quarters of the participating students (73% and 72% respectively) declared that they would have chosen the study condition ‘Friendly Feedback’ if given the opportunity. Among the students in the IVFF at both time points the percentage was 95%. Among the students in the CG, 20% at t5 and 29% at t8 declared that they would choose the control condition with a weekly mandatory examination. Also in this group, 11% at t5 and 14% at t8 would choose the IVSM. In the IVSM, 54% at t5 and 64% at t8 would choose the IVFF, and 33% at t5 and 27% at t8 would choose their current option (Table 1).

**Table 1:** Preferred condition of medical students at the end of winter (t5) / summer semester (t8; %)

	CG	IVSM	IVFF
**t5**			
Total	12.5	14.1	73.4
CG	20.0	11.1	68.9
IVSM	12.8	33.3	53.8
IVFF	0.0	4.5	95.5
**t8**			
Total	14.4	13.6	72.0
CG	28.6	14.3	57.1
IVSM	9.1	27.3	63.6
IVFF	2.3	2.3	95.3

CG: control group; IVSM: intervention stress management, IVFF: intervention friendly feedback

## Discussion

In this study in the setting of the dissection course we compared, using a randomized, controlled design, the effect of one intervention aimed at self-generated stress (IVSM) and one aimed at the stress-generating context (IVFF). Summarizing the results regarding our research question, we found that a significantly larger proportion of students in the ‘Friendly Feedback’ intervention group experienced less acute and chronic stress than in the control group. For the ‘Stress Management’ intervention group this was true in the second (summer) semester. Anxiety and negative affect were lower in students of the IVFF while positive affect, intrinsic motivation and self-efficacy were higher than in the CG. Self-perceived academic performance and preparation did not differ between the study groups.

### Stress during the dissection course

The dissection course in anatomy is unanimously described as a stressful experience in the initial years of medical education [51]. One source of stress, at which most published interventions are directed, is the issue of first contact with a dead corpse and the dissection of a human body [26, 52]. Another important factor is the vast amount of content to be learned and the common frequent testing. There is a distinct connection between these latter two factors because teachers themselves admit that they do not believe that intrinsic motivation alone would be enough to handle the material, but that examination has to give an additional impulse so that students really engage in the topic. Scott approves this notion when he writes: “As a medical educator who sits at curriculum tables, I often hear calls for testing to ensure that students ‘learn it’; after all, everyone knows that assessment drives learning. Doesn’t it?” [53]. In contrast, self-determination theory states that people are by nature active and engaged and are interested in fulfilling general psychological needs in terms of competence, autonomy, and relatedness [54].

### Intervention stress management and friendly feedback

The autonomy-supportive teaching style that was developed on the basis of SDT judges tests and examinations critically and emphasizes approaches that acknowledge students’ perspective and appreciates good performance. It has been shown that this teaching style reduces stress, increases intrinsic motivation and fosters conceptual learning [31]. In line with this and our first hypothesis, a significantly lower proportion of students in the ‘Friendly Feedback’ intervention group that comprised principles of autonomy-supportive teaching, perceived high stress levels, anxiety, and negative affect than among fellow students in the CG. Instead, a higher positive affect and self-efficacy were reported. Other studies confirmed that reduced stress, positive affect, and self-efficacy were closely related to high academic performance [17, 18]. It might indicate indirect support for the high stress levels found in the control group that (maybe compensatory) relaxation during the semester break was higher in the CG than in both intervention groups. The decreasing stress scores at t6 and t7 in the CG and in the IVSM group might be interpreted as a certain adaptation to the testing. However, these scores are still higher than those in the IVFF group and at t8 the scores rise again to the levels of the first semester.

In line with our second hypothesis, intrinsic motivation was higher in the IVFF than in the CG at all measurement points. As regards the decreasing intrinsic motivation in all study groups, it should be borne in mind that besides anatomy, several more topics had to be studied, adding to the vast amount to be learned, which may frustrate even the most ambitious students [55–57]. In contrast, for Brazilian medical students, a curriculum reform that reduced content and implemented elements of autonomy-supportive learning, increased intrinsic and extrinsic learning motivation [58].

Slightly in contrast to our first hypothesis, for students in the IVSM it was obviously difficult to apply the techniques of the mindset-oriented stress management training during the winter semester. However, in the summer semester and up to the oral and written examinations at the end of the semester, a lower proportion of the IVSM felt highly stressed than in the CG. This is in line with the results of a pilot study with the addisca training, which indicated that these effects were particular evident during periods of testing [30]. Perhaps the experience of stress related specifically to study is a prerequisite for effectively applying the course content. This was also supported by the fact that a higher percentage would choose this intervention again at t8 than at t5. Studies in the working population affirm that a stress-reducing effect was especially/only seen in those who presented with elevated stress scores at the beginning of the intervention [59, 60]. This was also true for self-efficacy [61].

An interesting result from our study was the fact that even after eliminating a major contextual factor of stress in the IVFF, a smaller but relevant proportion of students in this intervention group still felt severely stressed. It is obviously not only the concern about failing the examination that causes stress but might also be a fear of being seen in an unfavorable light in front of fellow students and teachers. This fear characterizes in particular maladaptive forms of perfectionism, which could be self-oriented, others-oriented or related to the judgment of others [22, 62]. For some of the students in the IVFF, these fears seemed to outweigh the relief provided by the omitted tests. Studies in medical students confirm that maladaptive perfectionism is associated with greater distress and symptoms of depression and hopelessness regarding academic performance [21]. This might be especially true for an extreme form of self-doubt known as ‘imposter phenomenon’ (IP). The IP is a form of distrust in one’s abilities and performance despite evidence of competence, success and positive feedback [63, 64]. It is important to note that this is not primarily a problem of underperforming or failing students. On the contrary, it often affects high-achieving individuals at all career stages [63]. Among medical students and residents proportions of 30−40% are reported, with women being affected to a greater extent than males [65, 66]. In contrast, adaptive forms of perfectionism were associated with positive affect and good performance [21].

### Academic performance

With regard to academic performance, on the one hand, there is a fear that without testing, students will not learn enough [53]; on the other hand, many studies show that testing causes anxiety, which in turn negatively affects academic performance [67, 68]. Contrary to the apprehensions of the teachers and in line with our third hypothesis, in our study, the perceived readiness of students for the written and oral exams was not significantly different between the intervention and control groups. However, it has to be noted that self-estimation by freshmen students may be flawed by inexperience and may differ significantly to the estimation of teachers or peers (Dunning–Kruger effect) [69–71]. Because even the standard examination, practiced in the CG and the IVSM, operated on a pass/fail condition (and approximately 95% of students pass the examination), it was not possible to correlate the study groups to objective performance parameters. We therefore intend to examine the correlation of the study group and the anatomy point score in the M1 exam in a consecutive study.

In the context of stress and academic performance, the question of how much stress could be or should be accepted or tolerated may be of interest. In view of the performance-enhancing effect of a certain physiological activation [72, 73], a no-stress policy probably seems neither realistic nor desirable. In the current study, a certain level of stress was accepted by teachers to promote intensive learning. However, the results of our study demonstrate that there might be options to reduce unnecessary high stress levels.

### Strengths and limitations

A major strength of our study was the randomized, controlled design and the high number of students, who were willing to participate. However, with three study groups and the slightly varying participation numbers per measuring point in the longitudinal design, the number of participants was too small to execute gender-specific analyses. To compare intervention versus non-intervention could be criticized because an effect might be too expected. However, we were particularly interested in a comparison of the two interventions with the standard procedure. With two interventions, there was also more than one option for comparison with the control. The study was only conducted at one university. However, considering comparable content and examination elements at other universities, the results appear to be at least partially applicable to other study locations. To date, we have only been able to measure performance via self-perception. Especially in freshmen students, this might be limited by inexperience (Dunning–Kruger effect, see above). It is planned to survey students after M1 and correlate the grades in the anatomy part of this examination as objective parameters with the intervention groups.

In summary it needs to be questioned, whether students at the beginning of their course of study have to be coerced to learn an overwhelming amount of content by the use of frequent examinations. On the basis of the SDT and autonomy-supportive learning that have been applied in the IVFF, an empowering approach seems to be much more promising, because it reduces stress and anxiety, fosters self-efficacy and intrinsic motivation and leads to at least similar results regarding perceived academic performance. Based on a review and meta-analysis, Richardson et al. [20] state that ‘teachers’ behaviors are likely to be important for boosting and maintaining students’ self-efficacy. Setting graded tasks, providing feedback on successful performance, and lowering students’ anxiety and stress about coursework, exams, and presentations promote mastery experiences and thereby increase self-efficacy’. They also suggest that interventions early in students’ university career may be most effective because the strongest correlates identified in their review, namely performance self-efficacy and grade goals, were likely to be more fluid during the early stages of skills development. [20].

## Conclusion

Replacing formal tests with friendly feedback has proven to be an effective measure to reduce stress and negative affect, and foster positive affect, self-efficacy, and intrinsic motivation, while it did not impair self-perceived academic performance. A review of performance based on objective criteria appears to be important for future studies. If this confirms the current findings, a regular offer of a revised stress management course and an adjustment of the course delivery with the aim of reducing unnecessary stress would appear appropriate.

### Electronic supplementary material

Below is the link to the electronic supplementary material.

Supplementary Material 1

Supplementary Material 2

## Data Availability

The data that support the findings of this study are available from the corresponding author upon reasonable request.
