# Supplementary material for: Test-reduced teaching for stimulation of intrinsic motivation (TRUST): a randomized controlled intervention study
Source: BMC Med Educ. 2024 Jul 3;24:718. doi: 10.1186/s12909-024-05640-7 (PMC11221006; doi:10.1186/s12909-024-05640-7)
Supplement: Supplementary file 1 — Supplementary Material 1 [file 12909_2024_5640_MOESM1_ESM.docx]

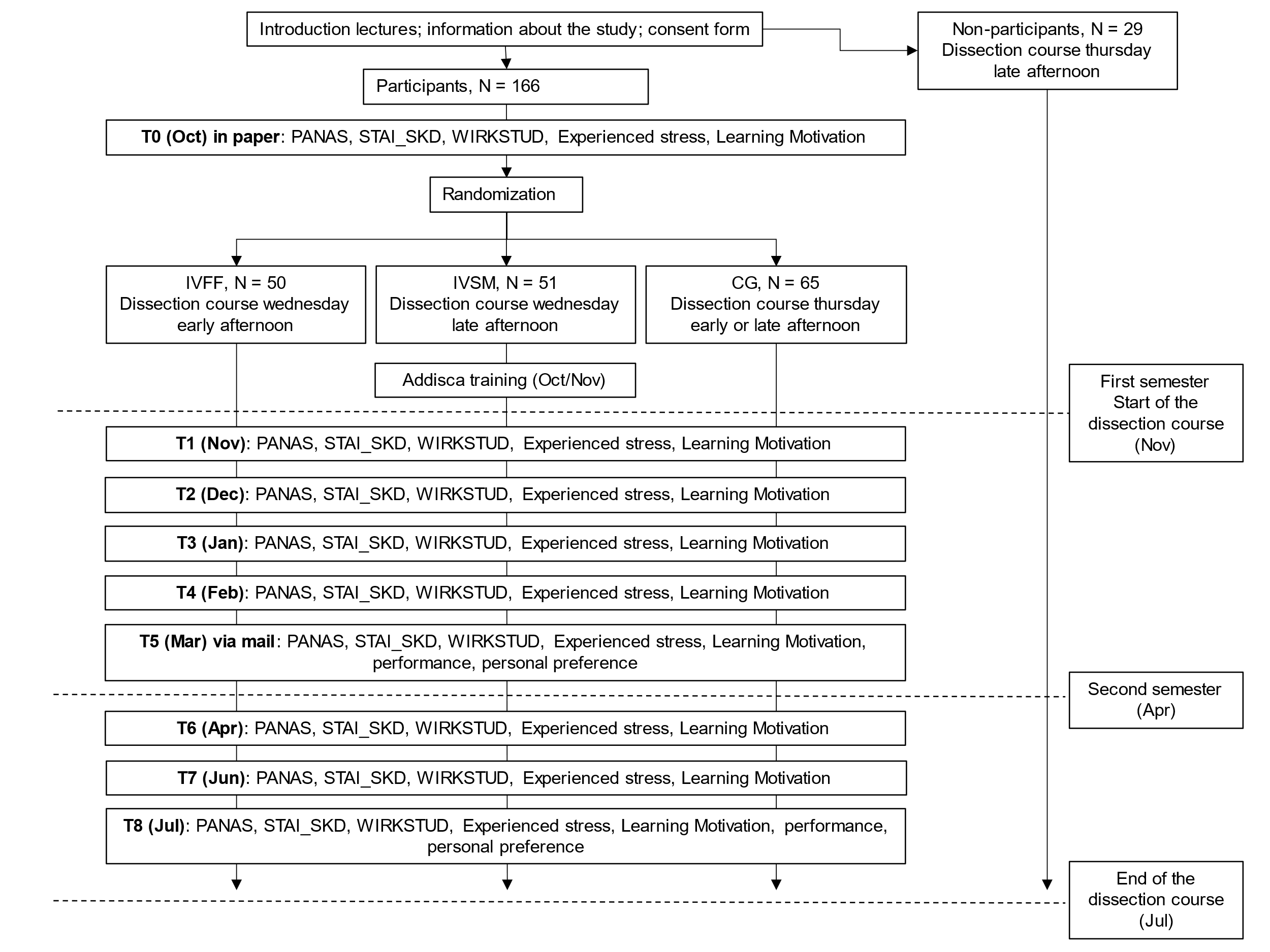


Additional file 1 Study design

CG: control group; IVSM: intervention stress management, IVFF: intervention friendly feedback; PANAS: Positive and Negative Affect Scales; STAI_SKD: State-Trait Anxiety Inventory; WIRKSTUD: Self-efficacy
