# Supplementary material for: Test-reduced teaching for stimulation of intrinsic motivation (TRUST): a randomized controlled intervention study
Source: BMC Med Educ. 2024 Jul 3;24:718. doi: 10.1186/s12909-024-05640-7 (PMC11221006; doi:10.1186/s12909-024-05640-7)
Supplement: Supplementary file 2 — Supplementary Material 2 [file 12909_2024_5640_MOESM2_ESM.docx]

**Additional file 2**

Post-hoc comparison results for outcome measures with a significant interaction effect (Stress, Anxiety, Self-efficacy, Positive and Negative Affect, and Intrinsic Motivation)

| Timepoint | Comparison | Estimate | *SE* | *df* | *t* | *p* | Significance |
| --- | --- | --- | --- | --- | --- | --- | --- |
| Stress |  |  |  |  |  |  |  |
| 0 | CG vs. IVSM | 0.21 | .37 | 636 | 0.57 | .839 |  |
| 0 | CG vs. IVFF | -0.22 | .36 | 617 | -0.60 | .823 |  |
| 0 | IVSM vs. IVFF | -0.42 | .39 | 631 | -1.09 | .521 |  |
| 1 | CG vs. IVSM | 0.45 | .37 | 626 | 1.23 | .438 |  |
| 1 | CG vs. IVFF | -0.04 | .36 | 621 | -0.12 | .992 |  |
| 1 | IVSM vs. IVFF | -0.49 | .39 | 619 | -1.28 | .410 |  |
| 2 | CG vs. IVSM | 0.35 | .38 | 673 | 0.92 | .628 |  |
| 2 | CG vs. IVFF | 0.97 | .36 | 632 | 2.67 | .021 | * |
| 2 | IVSM vs. IVFF | 0.63 | .39 | 641 | 1.61 | .242 |  |
| 3 | CG vs. IVSM | 0.33 | .38 | 667 | 0.87 | .658 |  |
| 3 | CG vs. IVFF | 1.43 | .37 | 646 | 3.88 | <.001 | *** |
| 3 | IVSM vs. IVFF | 1.10 | .39 | 656 | 2.79 | .015 | * |
| 4 | CG vs. IVSM | -0.08 | .38 | 687 | -0.21 | .977 |  |
| 4 | CG vs. IVFF | 1.36 | .38 | 680 | 3.64 | .001 | *** |
| 4 | IVSM vs. IVFF | 1.44 | .40 | 695 | 3.58 | .001 | ** |
| 5 | CG vs. IVSM | -0.30 | .40 | 774 | -0.75 | .734 |  |
| 5 | CG vs. IVFF | -0.52 | .39 | 744 | -1.33 | .378 |  |
| 5 | IVSM vs. IVFF | -0.22 | .41 | 719 | -0.54 | .853 |  |
| 6 | CG vs. IVSM | 0.68 | .42 | 839 | 1.63 | .233 |  |
| 6 | CG vs. IVFF | 1.04 | .39 | 766 | 2.65 | .022 | * |
| 6 | IVSM vs. IVFF | 0.37 | .43 | 788 | 0.87 | .662 |  |
| 7 | CG vs. IVSM | 0.69 | .41 | 803 | 1.69 | .211 |  |
| 7 | CG vs. IVFF | 0.72 | .39 | 733 | 1.87 | .150 |  |
| 7 | IVSM vs. IVFF | 0.04 | .40 | 698 | 0.09 | .996 |  |
| 8 | CG vs. IVSM | 0.68 | .40 | 790 | 1.69 | .210 |  |
| 8 | CG vs. IVFF | 0.77 | .38 | 720 | 1.99 | .115 |  |
| 8 | IVSM vs. IVFF | 0.08 | .42 | 755 | 0.20 | .978 |  |
| Anxiety |  |  |  |  |  |  |  |
| 0 | CG vs. IVSM | 0.08 | .13 | 377 | 0.63 | .805 |  |
| 0 | CG vs. IVFF | 0.07 | .13 | 366 | 0.50 | .873 |  |
| 0 | IVSM vs. IVFF | -0.02 | .14 | 374 | -0.13 | .991 |  |
| 1 | CG vs. IVSM | 0.13 | .13 | 372 | 0.99 | .587 |  |
| 1 | CG vs. IVFF | 0.11 | .13 | 369 | 0.83 | .687 |  |
| 1 | IVSM vs. IVFF | -0.02 | .14 | 368 | -0.16 | .986 |  |
| 2 | CG vs. IVSM | 0.02 | .13 | 396 | 0.15 | .987 |  |
| 2 | CG vs. IVFF | 0.63 | .13 | 374 | 4.83 | <.001 | *** |
| 2 | IVSM vs. IVFF | 0.61 | .14 | 378 | 4.38 | <.001 | *** |
| 3 | CG vs. IVSM | 0.24 | .13 | 396 | 1.81 | .166 |  |
| 3 | CG vs. IVFF | 0.70 | .13 | 388 | 5.27 | <.001 | *** |
| 3 | IVSM vs. IVFF | 0.45 | .14 | 391 | 3.22 | .004 | ** |
| 4 | CG vs. IVSM | 0.32 | .14 | 409 | 2.37 | .048 | * |
| 4 | CG vs. IVFF | 0.58 | .13 | 399 | 4.36 | <.001 | *** |
| 4 | IVSM vs. IVFF | 0.26 | .14 | 412 | 1.82 | .163 |  |
| 5 | CG vs. IVSM | 0.07 | .14 | 456 | 0.52 | .864 |  |
| 5 | CG vs. IVFF | 0.52 | .14 | 427 | 3.78 | <.001 | *** |
| 5 | IVSM vs. IVFF | 0.44 | .14 | 422 | 3.07 | .007 | ** |
| 6 | CG vs. IVSM | 0.31 | .14 | 491 | 2.14 | .082 |  |
| 6 | CG vs. IVFF | 0.53 | .14 | 448 | 3.85 | <.001 | *** |
| 6 | IVSM vs. IVFF | 0.23 | .15 | 465 | 1.51 | .285 |  |
| 7 | CG vs. IVSM | 0.27 | .14 | 471 | 1.94 | .129 |  |
| 7 | CG vs. IVFF | 0.51 | .14 | 433 | 3.72 | .001 | *** |
| 7 | IVSM vs. IVFF | 0.24 | .14 | 404 | 1.65 | .226 |  |
| 8 | CG vs. IVSM | 0.19 | .14 | 458 | 1.32 | .385 |  |
| 8 | CG vs. IVFF | 0.63 | .14 | 413 | 4.65 | <.001 | *** |
| 8 | IVSM vs. IVFF | 0.44 | .15 | 429 | 3.05 | .007 | ** |
| Self-efficacy |  |  |  |  |  |  |  |
| 0 | CG vs. IVSM | -0.10 | .65 | 367 | -0.16 | .987 |  |
| 0 | CG vs. IVFF | 0.03 | .64 | 353 | 0.05 | .999 |  |
| 0 | IVSM vs. IVFF | 0.13 | .69 | 356 | 0.20 | .979 |  |
| 1 | CG vs. IVSM | -1.23 | .65 | 351 | -1.91 | .137 |  |
| 1 | CG vs. IVFF | 0.13 | .64 | 351 | 0.20 | .978 |  |
| 1 | IVSM vs. IVFF | 1.36 | .68 | 350 | 2.00 | .114 |  |
| 2 | CG vs. IVSM | -0.99 | .66 | 374 | -1.50 | .291 |  |
| 2 | CG vs. IVFF | -1.67 | .64 | 355 | -2.60 | .027 | * |
| 2 | IVSM vs. IVFF | -0.68 | .68 | 356 | -1.00 | .579 |  |
| 3 | CG vs. IVSM | -0.89 | .66 | 371 | -1.37 | .360 |  |
| 3 | CG vs. IVFF | -2.05 | .65 | 360 | -3.17 | .005 | ** |
| 3 | IVSM vs. IVFF | -1.15 | .69 | 365 | -1.67 | .219 |  |
| 4 | CG vs. IVSM | -0.51 | .66 | 380 | -0.78 | .718 |  |
| 4 | CG vs. IVFF | -1.82 | .65 | 375 | -2.79 | .015 | * |
| 4 | IVSM vs. IVFF | -1.31 | .70 | 383 | -1.87 | .149 |  |
| 5 | CG vs. IVSM | -0.85 | .68 | 427 | -1.25 | .424 |  |
| 5 | CG vs. IVFF | -1.92 | .67 | 401 | -2.89 | .012 | * |
| 5 | IVSM vs. IVFF | -1.07 | .71 | 396 | -1.51 | .287 |  |
| 6 | CG vs. IVSM | -1.13 | .70 | 459 | -1.62 | .240 |  |
| 6 | CG vs. IVFF | -2.54 | .68 | 420 | -3.76 | <.001 | *** |
| 6 | IVSM vs. IVFF | -1.41 | .73 | 435 | -1.94 | .129 |  |
| 7 | CG vs. IVSM | -0.77 | .69 | 436 | -1.11 | .507 |  |
| 7 | CG vs. IVFF | -1.54 | .67 | 405 | -2.30 | .056 |  |
| 7 | IVSM vs. IVFF | -0.77 | .70 | 383 | -1.11 | .510 |  |
| 8 | CG vs. IVSM | -0.92 | .69 | 432 | -1.34 | .372 |  |
| 8 | CG vs. IVFF | -1.42 | .66 | 388 | -2.15 | .081 |  |
| 8 | IVSM vs. IVFF | -0.50 | .71 | 400 | -0.71 | .761 |  |
| Positive affect |  |  |  |  |  |  |  |
| 0 | CG vs. IVSM | 0.09 | .12 | 567 | 0.77 | .721 |  |
| 0 | CG vs. IVFF | 0.10 | .12 | 556 | 0.85 | .672 |  |
| 0 | IVSM vs. IVFF | 0.01 | .13 | 560 | 0.06 | .998 |  |
| 1 | CG vs. IVSM | 0.01 | .12 | 557 | 0.07 | .997 |  |
| 1 | CG vs. IVFF | -0.05 | .12 | 558 | -0.41 | .913 |  |
| 1 | IVSM vs. IVFF | -0.06 | .13 | 559 | -0.45 | .896 |  |
| 2 | CG vs. IVSM | 0.02 | .12 | 596 | 0.14 | .989 |  |
| 2 | CG vs. IVFF | -0.20 | .12 | 566 | -1.63 | .233 |  |
| 2 | IVSM vs. IVFF | -0.21 | .13 | 567 | -1.67 | .217 |  |
| 3 | CG vs. IVSM | 0.23 | .12 | 609 | 1.84 | .158 |  |
| 3 | CG vs. IVFF | -0.32 | .12 | 589 | -2.65 | .023 | * |
| 3 | IVSM vs. IVFF | -0.55 | .13 | 599 | -4.22 | <.001 | *** |
| 4 | CG vs. IVSM | 0.06 | .13 | 640 | 0.50 | .871 |  |
| 4 | CG vs. IVFF | -0.41 | .13 | 640 | -3.31 | .003 | ** |
| 4 | IVSM vs. IVFF | -0.48 | .13 | 657 | -3.54 | .001 | ** |
| 5 | CG vs. IVSM | 0.11 | .13 | 687 | 0.87 | .660 |  |
| 5 | CG vs. IVFF | 0.02 | .13 | 657 | 0.18 | .983 |  |
| 5 | IVSM vs. IVFF | -0.09 | .13 | 645 | -0.68 | .777 |  |
| 6 | CG vs. IVSM | 0.09 | .14 | 789 | 0.63 | .804 |  |
| 6 | CG vs. IVFF | -0.11 | .13 | 690 | -0.83 | .687 |  |
| 6 | IVSM vs. IVFF | -0.19 | .14 | 750 | -1.36 | .363 |  |
| 7 | CG vs. IVSM | -0.09 | .13 | 687 | -0.72 | .751 |  |
| 7 | CG vs. IVFF | -0.39 | .13 | 658 | -3.13 | .005 | ** |
| 7 | IVSM vs. IVFF | -0.30 | .13 | 589 | -2.34 | .051 |  |
| 8 | CG vs. IVSM | -0.02 | .13 | 708 | -0.17 | .984 |  |
| 8 | CG vs. IVFF | -0.43 | .12 | 632 | -3.45 | .002 | ** |
| 8 | IVSM vs. IVFF | -0.41 | .13 | 653 | -3.03 | .007 | ** |
| Negative affect |  |  |  |  |  |  |  |
| 0 | CG vs. IVSM | 0.02 | .12 | 408 | 0.16 | .987 |  |
| 0 | CG vs. IVFF | -0.02 | .12 | 399 | -0.15 | .988 |  |
| 0 | IVSM vs. IVFF | -0.04 | .13 | 403 | -0.29 | .955 |  |
| 1 | CG vs. IVSM | 0.12 | .12 | 395 | 0.98 | .592 |  |
| 1 | CG vs. IVFF | 0.03 | .12 | 391 | 0.29 | .954 |  |
| 1 | IVSM vs. IVFF | -0.08 | .12 | 393 | -0.65 | .793 |  |
| 2 | CG vs. IVSM | 0.06 | .12 | 422 | 0.54 | .852 |  |
| 2 | CG vs. IVFF | 0.46 | .12 | 403 | 3.91 | <.001 | *** |
| 2 | IVSM vs. IVFF | 0.39 | .12 | 400 | 3.17 | .005 | ** |
| 3 | CG vs. IVSM | 0.30 | .12 | 434 | 2.50 | .035 | * |
| 3 | CG vs. IVFF | 0.52 | .12 | 411 | 4.39 | <.001 | *** |
| 3 | IVSM vs. IVFF | 0.22 | .13 | 423 | 1.71 | .202 |  |
| 4 | CG vs. IVSM | 0.17 | .12 | 439 | 1.37 | .359 |  |
| 4 | CG vs. IVFF | 0.40 | .12 | 433 | 3.35 | .003 | ** |
| 4 | IVSM vs. IVFF | 0.24 | .13 | 438 | 1.85 | .156 |  |
| 5 | CG vs. IVSM | -0.04 | .13 | 481 | -0.32 | .945 |  |
| 5 | CG vs. IVFF | -0.07 | .12 | 455 | -0.58 | .834 |  |
| 5 | IVSM vs. IVFF | -0.03 | .13 | 448 | -0.23 | .971 |  |
| 6 | CG vs. IVSM | 0.22 | .13 | 530 | 1.70 | .206 |  |
| 6 | CG vs. IVFF | 0.27 | .12 | 464 | 2.23 | .067 |  |
| 6 | IVSM vs. IVFF | 0.06 | .13 | 500 | 0.41 | .911 |  |
| 7 | CG vs. IVSM | 0.04 | .13 | 486 | 0.29 | .954 |  |
| 7 | CG vs. IVFF | 0.19 | .12 | 473 | 1.52 | .280 |  |
| 7 | IVSM vs. IVFF | 0.15 | .13 | 422 | 1.19 | .457 |  |
| 8 | CG vs. IVSM | 0.16 | .13 | 490 | 1.29 | .401 |  |
| 8 | CG vs. IVFF | 0.43 | .12 | 443 | 3.53 | .001 | ** |
| 8 | IVSM vs. IVFF | 0.26 | .13 | 452 | 2.05 | .103 |  |
| Intrinsic motivation |  |  |  |  |  |  |  |
| 0 | CG vs. IVSM | -0.43 | .43 | 437 | -0.99 | .581 |  |
| 0 | CG vs. IVFF | -0.36 | .43 | 433 | -0.85 | .672 |  |
| 0 | IVSM vs. IVFF | 0.07 | .46 | 430 | 0.15 | .988 |  |
| 1 | CG vs. IVSM | -0.95 | .43 | 439 | -2.18 | .076 |  |
| 1 | CG vs. IVFF | -0.70 | .43 | 431 | -1.63 | .233 |  |
| 1 | IVSM vs. IVFF | 0.25 | .46 | 434 | 0.54 | .852 |  |
| 2 | CG vs. IVSM | -0.08 | .44 | 474 | -0.18 | .982 |  |
| 2 | CG vs. IVFF | -0.42 | .43 | 441 | -0.97 | .596 |  |
| 2 | IVSM vs. IVFF | -0.34 | .46 | 448 | -0.73 | .745 |  |
| 3 | CG vs. IVSM | -0.34 | .44 | 469 | -0.77 | .721 |  |
| 3 | CG vs. IVFF | -1.42 | .44 | 454 | -3.26 | .003 | ** |
| 3 | IVSM vs. IVFF | -1.08 | .46 | 455 | -2.32 | .054 |  |
| 4 | CG vs. IVSM | 0.11 | .45 | 482 | 0.25 | .968 |  |
| 4 | CG vs. IVFF | -1.23 | .44 | 470 | -2.79 | .015 | * |
| 4 | IVSM vs. IVFF | -1.34 | .47 | 486 | -2.82 | .014 | * |
| 5 | CG vs. IVSM | -0.95 | .46 | 539 | -2.05 | .103 |  |
| 5 | CG vs. IVFF | -0.40 | .45 | 516 | -0.87 | .657 |  |
| 5 | IVSM vs. IVFF | 0.55 | .48 | 497 | 1.16 | .480 |  |
| 6 | CG vs. IVSM | 0.06 | .48 | 603 | 0.11 | .993 |  |
| 6 | CG vs. IVFF | -0.42 | .46 | 536 | -0.92 | .626 |  |
| 6 | IVSM vs. IVFF | -0.48 | .50 | 557 | -0.97 | .599 |  |
| 7 | CG vs. IVSM | 0.03 | .48 | 581 | 0.07 | .998 |  |
| 7 | CG vs. IVFF | -0.53 | .45 | 513 | -1.18 | .468 |  |
| 7 | IVSM vs. IVFF | -0.56 | .48 | 496 | -1.18 | .466 |  |
| 8 | CG vs. IVSM | -0.60 | .47 | 559 | -1.28 | .408 |  |
| 8 | CG vs. IVFF | -1.15 | .45 | 498 | -2.57 | .028 | * |
| 8 | IVSM vs. IVFF | -0.55 | .49 | 531 | -1.13 | .496 |  |
